# Mass Spectrometric Analysis of Antigenic Determinant Glycans of Soybean Glycoprotein Gly m Bd 30K

**DOI:** 10.3390/molecules30173571

**Published:** 2025-08-31

**Authors:** Lingmei Li, Sidi Luo, You Wu, Xuefei Feng, Yuxin Ding, Yajuan Zhou

**Affiliations:** Luoyang Key Laboratory of Organic Functional Molecules, College of Food and Drug, Luoyang Normal University, Luoyang 471934, China; 19179748121@163.com (S.L.); 15121680623@163.com (Y.W.); fxfqc1314@163.com (X.F.); 18535017849@163.com (Y.D.); dingyu017@163.com (Y.Z.)

**Keywords:** Gly m Bd 30K, *N*-glycan, ESI-MS, MS/MS

## Abstract

Glycosylation of many proteins has been revealed to be closely related to food allergy, and screening and structural analysis of related glycoproteins are essential for studies in this important area. Gly m Bd 30K is one of the major allergens that exist in soybeans. *N*-Glycans of the Gly m Bd 30K influenced the immunoreactivity and antigen-presenting efficiency. In this paper, soybean allergen glycoprotein Gly m Bd 30K was used as the research object. It was separated and purified by the combination of isoelectric point and Sepharose CL-6B gel. The glycoprotein was analyzed and identified by SDS-PAGE and MALDI-TOF MS. The *N*-glycans of Gly m Bd 30K glycoprotein were released and labeled by a newly developed one-pot method, and qualitatively and quantitatively analyzed by ESI-MS^n^ and HILIC-UV-MS/MS. The results showed that the purity of Gly m Bd 30K glycoprotein was 95%, and the relative molecular mass was 33,923 Da. The Gly m Bd 30K glycoprotein contained a total of six kinds of glycans, including two types: oligo-mannose type (4.3%) and paucimannose type (95.7%). The paucimannose modified with core α-1,3-fucose and β-1,2-xylose accounted for 92.87%. This study provides quality-reliable materials for the follow-up study of glycan sensitization and also provides a theoretical basis for the in-depth study of the specificity and biological function of the antigenic determinant of Gly m Bd 30K glycoprotein.

## 1. Introduction

Proteins are crucial for maintaining human life and health [1]. Soybean protein is rich in content and widely used in food, but it is one of the eight major allergenic foods announced by the United Nations [2,3]. At present, there is no significant treatment for food allergies, and the main way to prevent the disease is by avoiding contact with allergens [4,5]. However, there are many types of soy products in life, so it is relatively difficult to avoid contact [2,6]. Therefore, in-depth and systematic research on the allergens in soybeans can provide a theoretical basis for soybean desensitization research.

Glycosylation of many proteins has been revealed to be closely related to food allergy, and screening and structural analysis of related glycoproteins are essential for studies in this important area [7,8]. However, the glycoforms of a few of the major glycoproteins have not been analyzed, and, since they are one of the major sources of protein for human foods, this restricts further in-depth investigations into soybean allergy [9,10,11,12]. The soybean Gly m Bd 30K protein was the most allergenic allergen, and more than 65% of patients carry respective IgE antibodies in the serum of soybean allergic patients [13,14,15,16]. Studies have shown that glycoproteins were the main allergenic protein substances in soybeans, and the main reason why plant glycoproteins cause allergic reactions was that the *N*-glycan modified with β1,2-xylose and α1,3-fucose, that are cross-reactive carbohydrate determinants (CCD), specifically binds to IgE antibodies in the body to cause allergic reactions [17,18,19,20,21,22,23]. However, current sensitization studies mainly focus on the protein part, and little attention has been paid to the role of *N*-glycans in sensitization. Therefore, studying glycoproteins containing *N*-glycan structures with core α1,3-fucose and β1,2-xylose is of great significance for sensitization research, which will lay a theoretical foundation for the study of the sensitization mechanism of Gly m Bd 30K glycoprotein.

Gly m Bd 30K was a low-abundance glycoprotein in soybean protein, with strong allergenicity and difficult separation and purification [24,25]. At present, gene recombination or complex purification methods are often used for preparation, which are difficult to operate and costly [15,26,27]. Here, this paper uses a simple isoelectric point precipitation and gel filtration method to quickly purify Gly m Bd 30K glycoprotein. Studies have shown that the ratio of fucose, mannose, N-acetylglucosamine, and xylose among *N*-glycans of Gly m Bd 30K glycoprotein was 1:3:2:1, but the type and structure of the glycan cannot be determined [26]. Therefore, this study qualitatively and quantitatively analyzed the *N*-glycans of the Gly m Bd 30K protein. This *N*-glycan information can provide a reference for the functional study of the glycan determinant of the Gly m Bd 30K allergen, and also bring new methods and ideas for reducing and eliminating soybean allergy.

## 2. Results and Discussion

### 2.1. Isolation, Purification, and Identification of Gly m Bd 30K

**Figure 1:**
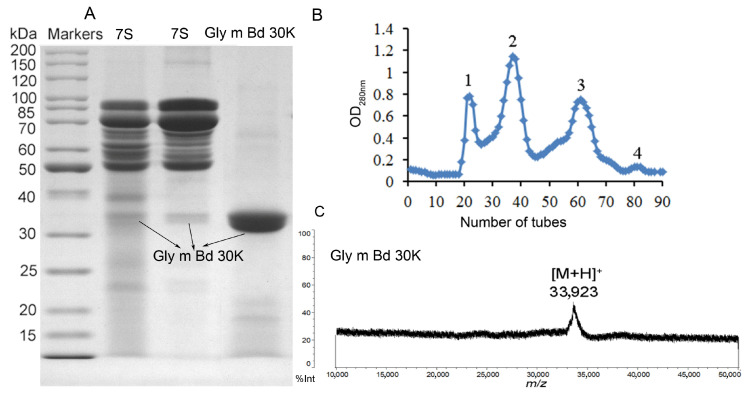
Profiling of eluted proteins by SDS-PAGE and MALDI-TOF MS. (**A**) SDS-PAGE of 7S globulin and Gly m Bd 30K. (**B**) Elution profile of Sepharose CL-6B gel filtration chromatography. The *x*-axis represents the number of tubes, and the *y*-axis represents the absorbance value at 280 nm (**C**) MALDI-TOF MS spectrum of Gly m Bd 30K.

In this paper, Gly m Bd 30K glycoprotein was purified by combining isoelectric point precipitation and gel chromatography. Sepharose CL-6B was selected as the material for separating proteins. This material has good toughness, is not easy to collapse, and has more advantages than dextran gel. Gly m Bd 30K protein was a low-abundance protein, and the previous purification methods have many steps and low yield [24]. This paper uses isoelectric point precipitation to obtain 7S globulin [28] and then uses Sepharose CL-6B gel filtration chromatography to purify Gly m Bd 30K glycoprotein. Only one gel filtration separation was needed to obtain high-purity Gly m Bd 30K glycoprotein. We used 10 g of soybean to extract the allergen Gly m Bd 30K, and the content of soybean protein isolate (SPI), 7S globulin, and Gly m Bd 30K were 3.18, 1.04, 0.08 g, and the extraction rate of Gly m Bd 30K was 0.8%. Four peaks appeared in the chromatography (Figure 1B), and Gly m Bd 30K were identified in peak 4 (Figure 1A). 11S globulin subunits and oil protein (Oleosin) were eluted in peak 3, peaks 1 and 2 were β-conglycinin (Figure S1), and the results were consistent with the references [29,30,31,32].

MALDI-TOF MS is an effective method for detecting protein molecular mass, is easy to operate, and can determine macromolecular proteins; compared with SDS-PAGE gel, MALDI-TOF MS can more accurately determine the protein molecular mass. Therefore, this paper uses SDS-PAGE to identify the purity of Gly m Bd 30K glycoprotein, and MALDI-TOF MS to determine its molecular mass. According to the SDS-PAGE analysis results (Figure 1A), we conducted two parallel experiments and found that there was a slight difference in the content of low-abundance proteins in the 7S globulin. But they all contain Gly m Bd 30K protein. We know 7S globulin were separated by many bands of globulin and includes the Gly m Bd 30K glycoprotein [24]. But purified Gly m Bd 30K has only one band, and its purity is 95% by gel gray scale analysis. Soybean Gly m Bd 30K, also known as P34, is a probable cysteine protease of the papain family and is highly conserved in all soybean families. P34 is a monomeric, insoluble, oil-body-associated glycoprotein with a molecular weight of 34 KDa [13,15,26,33,34,35]. Many references used a peptide-mass fingerprint to identify Gly m Bd 30K [35,36,37]. However, there are also reports in the literature that the molecular weight of Gly m Bd 30K was 30 KDa [6]. The Uniprot entry O64458 (Gly m Bd 30K) calculates the mass of the full-length protein with 43 KDa, and of the processed protein with 41 KDa, not taking into account the N-glycosylation. Therefore, there is controversy over the molecular weight of the protein in Gly m Bd 30K.

In this paper, identification of Gly m Bd 30K by intact mass analysis (Figure 1C) was performed in this study for the first time, according to the molecular mass of Gly m Bd 30K measured by MALDI-TOF MS (Figure 1C). Specifically, the ion at *m*/*z* 33,923 Da represents the Gly m Bd 30K, which was consistent with the literature reports [13,15,26,33,34,35,37]. But this result is different from that in Uniprot; the possible reason is that we used bovine serum albumin as the calibrator, and the glycan in proteins may also affect the results. Therefore, the analysis results of this paper have been verified by some of the literature. And it is provides reliable high-purity experimental materials for this study.

### 2.2. ESI-MS and MS/MS Analysis of Gly m Bd 30K Glycans

PNGase F and PNGase A have been widely used for the cleavage of *N*-glycans from glycoproteins, but PNGase F cannot release core α1,3-fucosylated *N*-glycans [38] and PNGase A was approximately inefficient to intact glycoproteins [39,40]. However, our laboratory developed a new chemical method for nonreductive *N*-glycan release and simultaneous labeling with PMP in one pot, which has no selectivity to different types of *N*-glycans [41]. The new PMP derivatization method is that which directly labels 2PMP on *N*-glycans after alkali hydrolysis of glycosidic bonds with NaOH (one-pot method).

**Figure 2:**
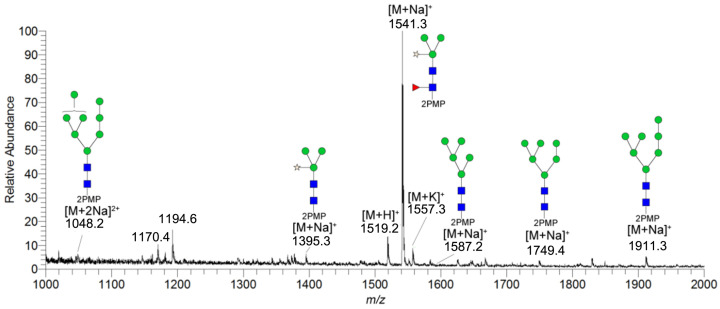
ESI-MS profiles of the *N*-glycans released from Gly m Bd 30K. Symbol nomenclature: 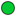, mannose; 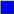, *N*-acetylglucosamine; 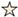, xylose; and 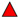, fucose.

In this paper, the “one-pot method” was used to release the *N*-glycans of Gly m Bd 30K glycoprotein and carry out ESI-MS^n^ analysis [41]. A total of six PMP-labeled glycans were obtained from Gly m Bd 30K (Figure 2). Most of the molecular ion peaks were assigned to [M + Na]^+^, although a few peaks were assigned to [M + H]^+^, [M + K]^+^ and [M + 2Na]^2+^. Among them, the ion at *m*/*z* 1911.3 ([M + Na]^+^) Da represent the structure Man_7_GlcNAc_2_(PMP)_2_; the ion at *m*/*z* 1749.4 ([M + Na]^+^) Da represent the structure Man_6_GlcNAc_2_(PMP)_2_; the ion at *m*/*z* 1587.2 ([M + Na]^+^) Da represent the structure Man_5_GlcNAc_2_(PMP)_2_; the ion at *m*/*z* 1557.3 ([M + K]^+^) Da, 1541.3 ([M + Na]^+^) Da, and 1519.2 ([M + H]^+^) Da represent the structure Man_3_FucXylG1cNAc_2_(PMP)_2_; the ion at *m*/*z* 1395.3 ([M + Na]^+^) Da represent the structure Man_3_XylG1cNAc_2_(PMP)_2_; the ion at *m*/*z* 1194.6 Da and 1170.4 Da represent the impurities; the ion at *m*/*z* 1048.2([M + 2Na]^2+^) represent Man_8_GlcNAc_2_(PMP)_2_. Of these *N*-glycans, Man_3_FucXylG1cNAc_2_(PMP)_2_ at *m*/*z* 1541.3 theoretically has two possible isomers, namely, core α1,3- and core α1,6-fucosylated *N*-glycan structures. It is already known that PNGase F is ineffective to core α1,3-fucosylated *N*-glycans, while the one-pot chemical method has no selectivity to both isomers [38,41]. Therefore, the two methods were employed here to differentiate α1,3- and core α1,6-fucosylated *N*-glycans. The *N*-glycan at *m*/*z* 1541.3 was not observed in the 7S globulin *N*-glycans released by PNGaseF but in those obtained by the one-pot chemical method, demonstrating that this glycan has only core α1,3-fucosylation modification (Figure S2).

All PMP-labeled glycans are identified by MS/MS (Figure S3), and the above glycans include four oligomannose type glycans and two paucimannose glycans. In addition, Bando N et al. [19] found that Gly m Bd 30K glycoprotein only contains glycan of structure Man_3_FucXylG1cNAc_2_; while the results of this study show that in addition to glycan of Man_3_FucXylG1cNAc_2_ structure type, Gly m Bd 30K also has a β1,2-xylose modified glycan structure Man_3_XylG1cNAc_2_ and high-mannose glycan structures Man_8_GlcNAc_2_(PMP)_2_, Man_7_GlcNAc_2_(PMP)_2_, Man_6_GlcNAc_2_(PMP)_2_, and Man_5_GlcNAc_2_(PMP)_2_. The ion at *m*/*z* 1541.3 ([M + Na]^+^) was the highest, indicating that the glycan is modified with β1,2-xylose and α1,3-fucose content in Gly m Bd 30K glycoprotein, and it is an important binding site for immune response. Therefore, the Gly m Bd 30K glycoprotein contains six *N*-glycans, and five *N*-glycans were newly discovered glycans; this might be due to the improved analysis method. However, the Gly m Bd 30K sample has a purity of only 95%, and the 5% of contaminants could be the source of the additional *N*-glycans. Furthermore, high-mannose *N*-glycans and the Man_3_XylG1cNAc_2_ glycan indicate incomplete processing of the *N*-glycans. The large number of core complex glycans may be the main reason for the strong allergic reaction caused by Gly m Bd 30K glycoprotein.

### 2.3. Separation and Quantitative Analysis of Gly m Bd 30K N-Glycans by Online HILIC-ESI-MS/MS

**Figure 3:**
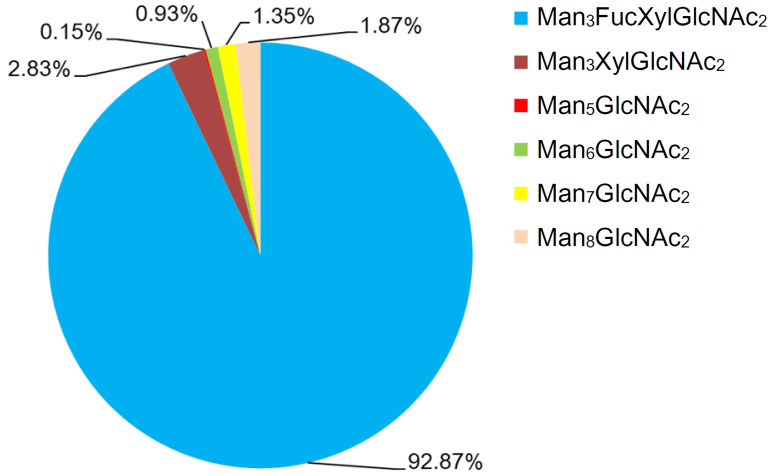
Chart showing the quantitative distribution of the *N*-glycans of Gly m Bd 30K. Notes: Man, mannose; GlcNAc, *N*-acetylglucosamine; Xyl, xylose; and Fuc, fucose.

Isomers exist extensively within the glycans released from glycoprotein. Online HILIC-UV-MS/MS^n^ analysis of the *N*-glycans derived from Gly m Bd 30K was performed to separate possible *N*-glycan isomers and obtain information about their distributions in abundance. The obtained extracted ion chromatograms (EICs) of these *N*-glycans are shown in Figure S4. Their qualitative information is summarized in Table 1, and quantitative information is in Figure 3.

As presented in Figure S3, the online HILIC-ESI-MS EICs give good profiling for the *N*-glycoforms of Gly m Bd 30K glycoprotein, especially for the possibly existing isomers of these *N*-glycans. Briefly, six peaks (*m*/*z* 1395.3, 1541.3, 1587.2, 1749.3, 1911.3, and 1048.3) were found in Gly m Bd 30K. The observed peaks of the six *N*-glycans at the ion *m*/*z* 1395.33, 1541.33, 1587.25, 1749.33, 1911.42, and 1048.33 occur at 36.50, 54.29, 53.28, 63.57, 73.62, and 82.94 min, respectively. Obviously, no glycan isomers were found in all of Gly m Bd 30K glycoprotein samples.

In order to characterize the quantitative distribution of different types of *N*-glycans for Gly m Bd 30K, their occupancy rates in the total *N*-glycans of gly m Bd 30K are summarized in Figure 3, based on the integral area of their UV peaks of HPLC. Obviously, the Gly m Bd 30K contains oligomannose type (4.3%) and paucimannose (95.7%) *N*-glycans. The most abundant *N*-glycan was Man_3_FucXylG1cNAc_2_ (92.87%), and the other *N*-glycans did not exceed 5%. Studies have shown that Gly m Bd 30K protein only contains Man_3_FucXylG1cNAc_2_ glycan [37], but this study identified five new glycans of the Gly m Bd 30K glycoprotein, and the proportion of these paucimannose was 7.13%.

Studies have found that, compared with mammalian protein glycans, plant proteins contain glycans modified with β1,2-xylose and core α1,3-fucose [42]. Therefore, the glycan structure of plant glycoproteins may have antigenic activity to animal cells. Studies have shown that glycoproteins with glycans modified with β1,2-xylose and α1,3-fucose were important epitopes recognized by IgE in allergic patients [43]. Therefore, it can be inferred that *N*-glycans modified with β1,2-xylose and α1,3-fucose were closely related to the allergic reaction of allergic patients and the Man_3_FucXylG1cNAc_2_ structure was the allergic component. This study found that the Man_3_FucXylG1cNAc_2_ structure was highly present on Gly m Bd 30K, which may be an important reason for the allergic reaction of Gly m Bd 30K glycoprotein in humans.

## 3. Materials and Methods

### 3.1. Reagents and Instruments

Soybeans *(Glycine max* L.) purchased from Xi’an Farmers’ Market (Xi’an, China); bovine serum albumin was purchased from Sigma Aldrich Co. (St. Louis, MO, USA). MW markers of proteins were from Fermentas (Burlington, Canada); Peptide: N-glycosidase F (PNGase F) was the product of New England BioLabs (Ipswich, MA, USA). α-cyano-4-hydroxy-cinnamic acid (CHCA), Sinapic acid (SA), Sodium dodecyl sulfate (SDS), 1-phenyl-3-methyl-5-pyrazolinone (PMP), β-mercaptoethanol, and Trifluoroacetic acid (TFA) was purchased from Shanghai aladdin company (Shanghai, China); Other reagents are analytical pure. The MD34 (retention MW: 8000–14,000) dialysis membrane was from Union Carbide Co., (Danbury, CT, USA); nonporous graphitized carbon (Carbograph) solid-phase extraction (SPE) columns (150 mg/4 mL) were purchased from Alltech Associates (Deerfield, IL, USA); Sepharose CL-6B packing (1 L) was purchased from General Electric Company (Fairfield, CT, USA); chromatography columns without filler material (2.5 cm × 1.2 m) were purchased from Beijing wan jing Bo mei Glass Products Co., Ltd. (Beijing, China); C18 solid phase extraction columns were purchased from Sigma Aldrich Co., (St. Louis, MO, USA); the experimental water was prepared in a Milli-Q system (Millipore, Burlington, MA, USA); electrospray ionization mass spectrometry (ESI-MS) and high performance liquid chromatograph (HPLC) were produced by Thermo scientific (Waltham, MA,, USA). AXIMA performance MALDI-TOF-MS was produced by Shimadzu (Tokyo, Japan). TSK-GEL amide-80 column (4.6 mm × 250 mm, 5 μm) was purchased from Tosoh Company (Tokyo, Japan).

### 3.2. Packing of Chromatography Column

Before using the gel, Sepharose CL-6B swelled in the 35mmol/L phosphate buffer (2.6 mmol/L NaH_2_PO_4_, 32.5 mmol/L Na2HPO4, 0.4 mol/L NaCl, pH 7.6) for one day. Then, the monomers, powders, and impurities in the gel were removed by water flotation, and the bubbles in the Sepharose CL-6B gel were expelled by a vacuum pump. Fix the column vertically, add a small amount of phosphate buffer phase to exclude the bottom gas of the column, and add some mobile phase to about 1/4 of the height of the column. Connect a funnel to the top of the column, with a neck diameter of about half of the column neck. Under stirring, slowly, uniformly, and continuously add the degassed gel suspension, while opening the outlet of the chromatography column to maintain an appropriate flow rate. The Sepharose CL-6B gel particles will rise layer by layer in a horizontal manner and deposit uniformly in the column until they reach the desired height. Finally, remove the funnel and cover the surface of the gel bed with a small piece of filter paper. Wash the Sepharose CL-6B gel bed with a large amount of eluent for a period of time.

### 3.3. Isolation and Purification of Gly m Bd 30K

Soybeans were grounded with a freeze-grinding machine and sifted through a 60-mesh sieve, then petroleum ether was used to remove fat four times. Defatted soybean powder was dissolved at a solid-to-liquid ratio of 1:15 (*m*/*V*) in 0.03 M Tris-HCl (pH 8.0) buffer and stirred at room temperature for 1 h. The total protein in the soybean was dissolved in the Tris-HCl, and centrifuged for 30 min (9000× *g*, 4 °C) to obtain supernatant. Then, add sodium bisulfite to a concentration of 0.01 mol/L based on the volume of supernatant. Adjust the pH to 6.4 with HCl (2 mol/L), then the mixture was stored at 4 °C overnight and centrifuged at 20 min (6500× *g*, 4 °C). After removing the sediment, NaCl was added to the supernatant to achieve a final concentration of NaCl that was 0.25 mol/L; adjust the pH 5.5 with HCl (2 mol/L). The mixture was stirred at room temperature for 30 min and then centrifuged (9000× *g*, 4 °C) for 30 min to remove the sediment. After that, add pre-cooled distilled water to dilute the supernatant to twice its original volume, and then adjust to pH 4.8 by HCl (2 mol/L). The mixture was stirred at 4 °C for 30 min and then centrifuged (9000× *g*, 4 °C) for 30 min. The sediment protein was soybean 7S globulin.

The 7S globulin was subjected to Sepharose CL-6B gel filtration chromatography to obtain Gly m Bd 30K [44]. Soybean 7S globulin was dissolved in a volume of minimum 35 mmol/L phosphate buffer (2.6 mmol/L NaH_2_PO_4_, 32.5 mmol/L Na_2_HPO_4_, 0.4 mol/L NaCl, pH 7.6) at a ratio of 1:5 (*m*/*V*), and then put on a Sepharose CL-6B column (2.5 cm × 1.2 m) that was previously equilibrated with 35 mmol/L phosphate buffer, and the proteins were eluted with the same buffer. The flow rate of elute buffer was 0.15 mL/min; Gly m Bd 30K was eluted by the same phosphate buffer and collected in the receptor machine. The absorbance of the protein solution was measured at 280 nm using ultraviolet spectrophotometer, and the elution curve was made with the number of tubes on the *x*-axis and the absorbance on the *y*-axis. Each elution peak was collected, and the separated peaks were subjected to 4 °C distilled water dialysis for desalination using the MD34 (MW: 8000–14,000) dialysis membrane for 3 days. It was then lyophilized and stored at −20 °C.

### 3.4. Identification of Gly m Bd 30K by SDS-PAGE

Sodium dodecyl sulfate-polyacrylamide gel (SDS-PAGE) was performed by Laemmli [45] by using 5% acrylamide in stacking gels and 12% acrylamide in separating gels. Gly m Bd 30K glycoprotein samples were dissolved in loading buffer (1% SDS, 40%glycerol, 0.1% dithiothreitol, and 0.05% bromophenol blue in 10 mM Tris-HCl buffer, pH 8.0) at the concentration of 2 mg/mL, and then heated at 100 °C for 10 min and cooled down afterwards. For a maximum visualization of Gly m Bd 30K, the amount of protein loaded was 10 μg for all samples. SDS-PAGE molecular weight standards ranging from 15 to 200 kDa were used. Electrophoresis was carried out at 90 V in the stacking gel and 120 V in the separating gel using a Tris-Glycine electrophoretic buffer containing 0.1% SDS (pH 8.3). The gel was stained for 1 h with 1% Coomassie Blue G250 dissolved in a mixture containing 45% methanol, 45% water, and 10% acetic acid, and destained with a mixture containing 87.5% water, 5% methanol, and 7.5% acetic acid. The gels were scanned using Im Bio-Rad GelDoc 2000 gel imaging and assessed by Gel-Pro Analyzer.

### 3.5. MALDI-TOF Analysis

MALDI-TOF-MS was performed on an AXIMA Performance instrument (Shimadzu, Tokyo, Japan). The MS data acquisition was carried out in positive ion linear mode, using an anitrogen laser with the power level set at 98 and a matrix of 10 mg/mL α-cyano-4-hydroxy-cinnamic acid (CHCA) prepared in 50% acetonitrile containing 0.1% TFA. Two shots accumulated for each profile were acquired for each sample spot. Two milligrams of purified Gly m Bd 30K were dissolved in 100 μL of deionized water, and 1 μL of the sample solution was mixed with 1 μL of matrix solution prior to spotting on a stainless steel target plate and drying at room temperature for analysis. We used the Bovine Serum Albumin (BSA) as standard proteins for calibration. The instrument was externally calibrated with 1 p mol Bovine Serum Albumin (BSA) on target and SA as MALDI matrix to calibration. All MALDI-TOF-MS spectra acquired represent the accumulation of just 100–150 single and unselected consecutive laser shots and are smoothed via a Gaussian algorithm. In all shown figures, the millivolt (mV) value of the base peak in the shown *m*/*z* range is given. For the evaluation of the signal-to-noise (S/N) ratio, the same smooth filter width of 100 was used at all times. All analyses were performed using the Shimadzu Biotech launchpad software (version 2.7.3).

### 3.6. Release of N-Glycans from 7S Globulin by PNGase F

7S globulin (2–5 mg) was dissolved in protein denaturing solution (500 μL) containing 5% SDS and 0.4 M DTT, and incubated at 100 °C for 10 min. When the sample was cooled, 50 μL of sodium phosphate buffer (0.5 mol/L, pH 7.5), 50 μL of 10% aqueous NP-40, and 1 μL of PNGase F (500 units) solution were sequentially added, followed by incubation at 37 °C for 24 h. Subsequently, the sample was boiled for 10 min to terminate the enzymatic reaction and then loaded onto a Sep-pak C18 SPE column. Elution of *N*-glycans was performed with 15 mL of water. Desalting of *N*-glycans was achieved using a Carbograph SPE column. The column was washed with 3 mL of water to remove salts, and the *N*-glycans were eluted with 25% aqueous acetonitrile solution. The eluates were concentrated to dryness under reduced pressure for further use.

### 3.7. Preparation of N-Glycans by One-Pot Method

The operation was performed according to a method developed in our laboratory [44]. Briefly, 2–5 mg of Gly m Bd 30K glycoprotein was suspended in 4 mL of 0.5 M aqueous NaOH solution containing 0.7 M PMP prior to the addition of 2 mL of methanol to the sample. The obtained mixture was incubated at 75 °C for 32 h. When the reactions were completed, the sample was neutralized with glacial acetic acid and washed three times with 4 mL of dichloromethane to remove excess PMP. The obtained sample solution was concentrated to dryness to remove methanol and excess acetic acid. Finally, the sample was redissolved in 1mL of water and loaded onto a Sep-Pak C18 SPE column for purification. The column was washed with 6 mL of water to remove salts and the PMP derivatives of *N*-glycans were eluted with 3 mL of 25% acetonitrile. The same method was used for the PMP labels of 7S globulin *N*-glycan released by PNGase F. The eluates were concentrated to dryness and stored at −20 °C for further analysis [46].

### 3.8. Analysis by ESI-MS and MS/MS

The ESI-MS analysis of glycans was performed on an LTQ XL linear ion trap mass spectrometer coupled with an electrospray ion source and an HPLC system (Thermo Scientific, Waltham, MA, USA). A total of 2–5 mg Gly m Bd 30K glycoprotein-released *N*-glycan (about 0.09 μg) was dissolved in 500 μL of 50% aqueous methanol (*vol*/*vol*) and directly infused into the mass spectrometer via a 2-μL Rheodyne loop. The infused glycans were brought into the electrospray ion source by a stream of 50% methanol at a flow rate of 20 μL/min. The voltage was 4 KV, and the sheath gas (N^2^) flow rate was 20.0 arb; the auxiliary gas (N^2^) flow rate was 10.0 arb, the capillary lens voltage was 250 V, and the capillary voltage and temperatures were 350 V and 300 °C. For the analysis by tandem mass spectrometry (MS/MS), the glycans were subjected to fragmentation by collision-induced dissociation (CID), with helium (He) as the collision gas. Collision parameters were left at default values with a normalized collision energy degree of 30 and an isotope width of *m*/*z* 3.00. The activation Q was set at 0.25 and the activation time at 30 ms. The MS and MS/MS data were recorded using the LTQ Tune software (version 2.7.0). The structure of each glycan was assigned according to MS/MS data combined with computational analysis using the GlycoWorkbench (version 1.1) software and knowledge of *N*-glycan biosynthesis [42].

### 3.9. Online HILIC-UV-MS/MS Analysis

Online HILIC-UV-MS/MS analysis of PMP-labeled glycans was also performed on the HPLC-ESI-MS system (Thermo scientific, Waltham, MA, USA) using a TSK-GEL amide-80 column (4.6 mm × 250 mm, 5 μm) (Tosoh Corporation, Tokyo, Japan). A total of 2–5 mg Gly m Bd 30K glycoprotein-released *N*-glycan (about 0.09 μg) was dissolved in 20 μL of 50% aqueous methanol, and 10 μL of the sample solution was injected by an autosampler. The elution gradient was as follows: solvent A, acetonitrile; solvent B, 10 mmol/L aqueous ammonium acetate (pH 6.0); time = 0–120 min, 80–60% A, 20–40% B, and 1 mL/min. The fractions eluted from the chromatographic column were directly imported into the ESI-MS system for detection. For the ESI-MS system, a rapid alteration mode between the segments of positive ion mode MS and MS-dependent MS/MS was adopted. For the MS-dependent MS/MS, the normalized collision energy was set at 30, and the lowest signal intensity was set at 500. The other parameters used for MS and MS-dependent MS/MS were the same as those described above. Data acquisition was performed using Xcalibur (version 2.2.42) software (Thermo). The obtained data were manually interpreted, and the proposed glycans compositions and sequences were checked using GlycoWorkbench software. The area values of chromatographic peaks of glycans were used for glycan quantification.

## 4. Conclusions

This is the first study that used the combination of isoelectric point precipitation and gel permeation chromatography to separate and purify the soybean allergenic glycoprotein Gly m Bd 30K. We analyzed the glycan structure and relative quantitative information of glycans by ESI-MS/MS^2^ and Online HILIC-UV-MS/MS. The results showed that the Gly m Bd 30K protein purity was 95%, and the relative molecular mass was 33,923 Da; the Gly m Bd 30K protein contained six kinds of *N*-glycans, including two types: oligomannose type (4.3%) and paucimannose (95.7%), and the Man_3_FucXylG1cNAc_2_ *N*-glycan related to soybean allergy accounted for a relatively high proportion of 92.87%. The large number of core complex glycans may be the main reason for the strong allergic reaction caused by Gly m Bd 30K protein. This study not only provides a material preparation method for glycan sensitization research but also provides a theoretical basis for a deeper understanding of the specificity and biological function of the antigenic epitope of Gly m Bd 30K.

## Figures and Tables

**Table 1:** Summary of the Information of Gly Bd 30K *N*-Glycans Obtained from Comprehensive Analysis by ESI-MS and Online HILIC-MS/MS.

*m*/*z*	Mass	Ion Type	Monosaccharide Composition ^a^	Proposed Structure ^b^	Retention Time (Min)
1395.3	1371.3	[M+Na]^+^	Man_3_XylG1cNA_2_ (PMP)_2_	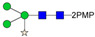	36.50
1519.2 1541.3 1557.3	1518.2 1518.3 1518.3	[M + H]^+^ [M + Na]^+^ [M + K]^+^	Man_3_FucXyl G1cNAc_2_(PMP)_2_	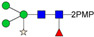	54.29
1587.2	1564.2	[M + Na]^+^	Man_5_GlcNAc_2_ (PMP)_2_	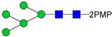	53.28
1749.4	1726.4	[M + Na]^+^	Man_6_GlcNAc_2_ (PMP)_2_	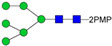	63.57
1911.3	1888.3	[M + Na]^+^	Man_7_GlcNAc_2_ (PMP)_2_	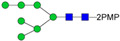	73.62
1048.3	2050.3	[M + 2Na]^2+^	Man_8_GlcNAc_2_ (PMP)_2_	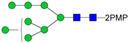	82.94

^a^ Man, mannose; GlcNAc, *N*-acetylhexosamine; Xyl, xylose; and Fuc, fucose. ^b^ Symbol nomenclature: 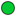, mannose; 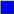, *N*-acetylglucosamine; 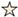, xylose; and 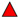, fucose.

## Data Availability

The data presented in this study are available on request from the corresponding author.

## Supplementary Materials

The following supporting information can be downloaded at the following address: https://www.mdpi.com/article/10.3390/molecules30173571/s1. Figure S1: Gly m Bd 30K protein was determined by SDS-PAGE; Markers were standard protein; SPI was soybean protein isolate; 7S was 7S globulin; peak 1, peak 2, peak 3, and peak 4 correspond to peaks 1–4 in Figure 1B, respectively. Peak 1 and 2 were β-conglycinin; peak 3 was 11S globulin subunits and oil protein (Oleosin); peak 4 was Gly m Bd 30K. Figure S2: EICs of Gly m Bd 30K N-glycans; Symbol nomenclature: , mannose; , N-acetylglucosamine; , xylose; and , fucose. Figure S3: MS/MS analysis of N-glycans released from Gly Bd 30K; , N-acetylglucosamine; , mannose; , fucose; and , xylose.
