# Supplementary material for: Mass Spectrometric Analysis of Antigenic Determinant Glycans of Soybean Glycoprotein Gly m Bd 30K
Source: Molecules. 2025 Aug 31;30(17):3571. doi: 10.3390/molecules30173571 (PMC12430132; doi:10.3390/molecules30173571)
Supplement: Supplementary file 1 [file molecules-30-03571-s001.zip › molecules-3740366-supplementary.pdf]

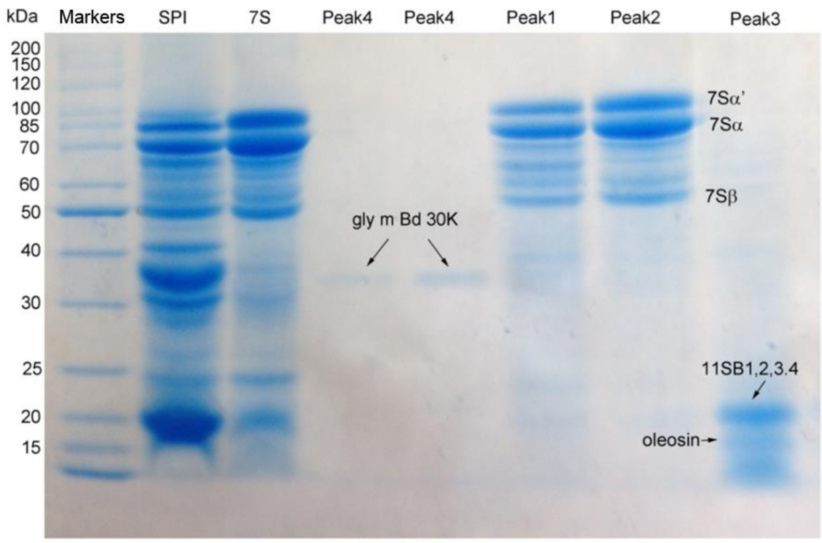

Figure S1 Profiling of eluted proteins by SDS-PAGE

Notes：Markers are standard protein; SPI is soybean protein isolate; 7S is 7S globulin; peak1-4 in FirureS1 correspond to peaks1-4 in figure 1B, respectively. Peak 1 and 2 were β-conglycinin; Peak 3 was 11S globulin subunits and oil protein (Oleosin); peak 4 was Gly m Bd 30K.

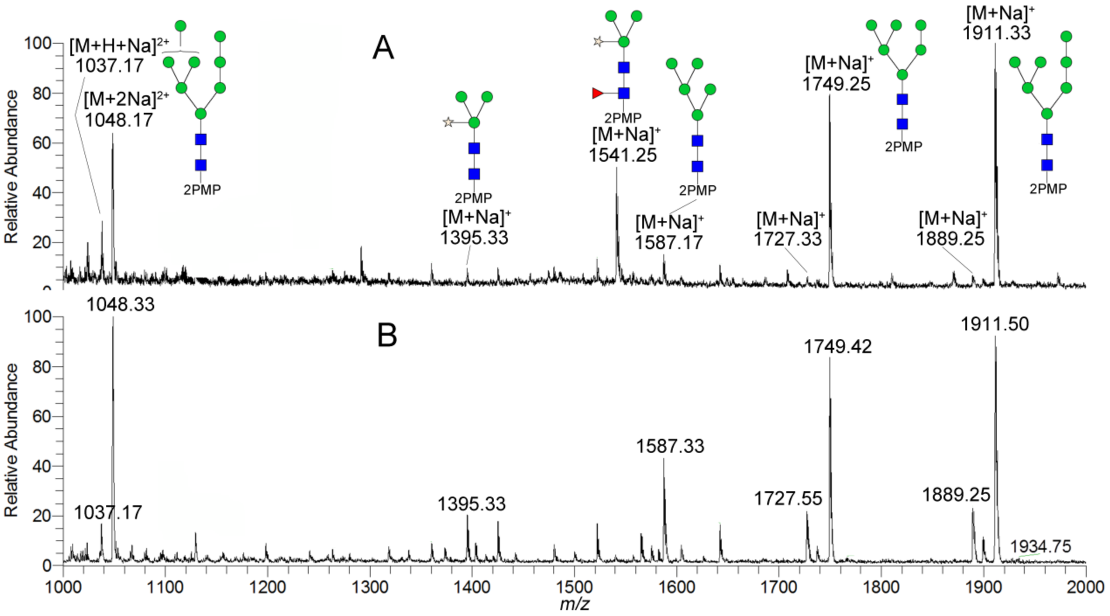

Figure S2 Representative ESI-MS profiles of 7S globulin *N*-glycans released by the one-pot chemical method (A) and PNGaseF combined with separated PMP labeling (B). Symbol nomenclature: ■, *N*-acetylglucosamine; red triangles, ▲, fucose; ☆, xylose; ●, mannose.

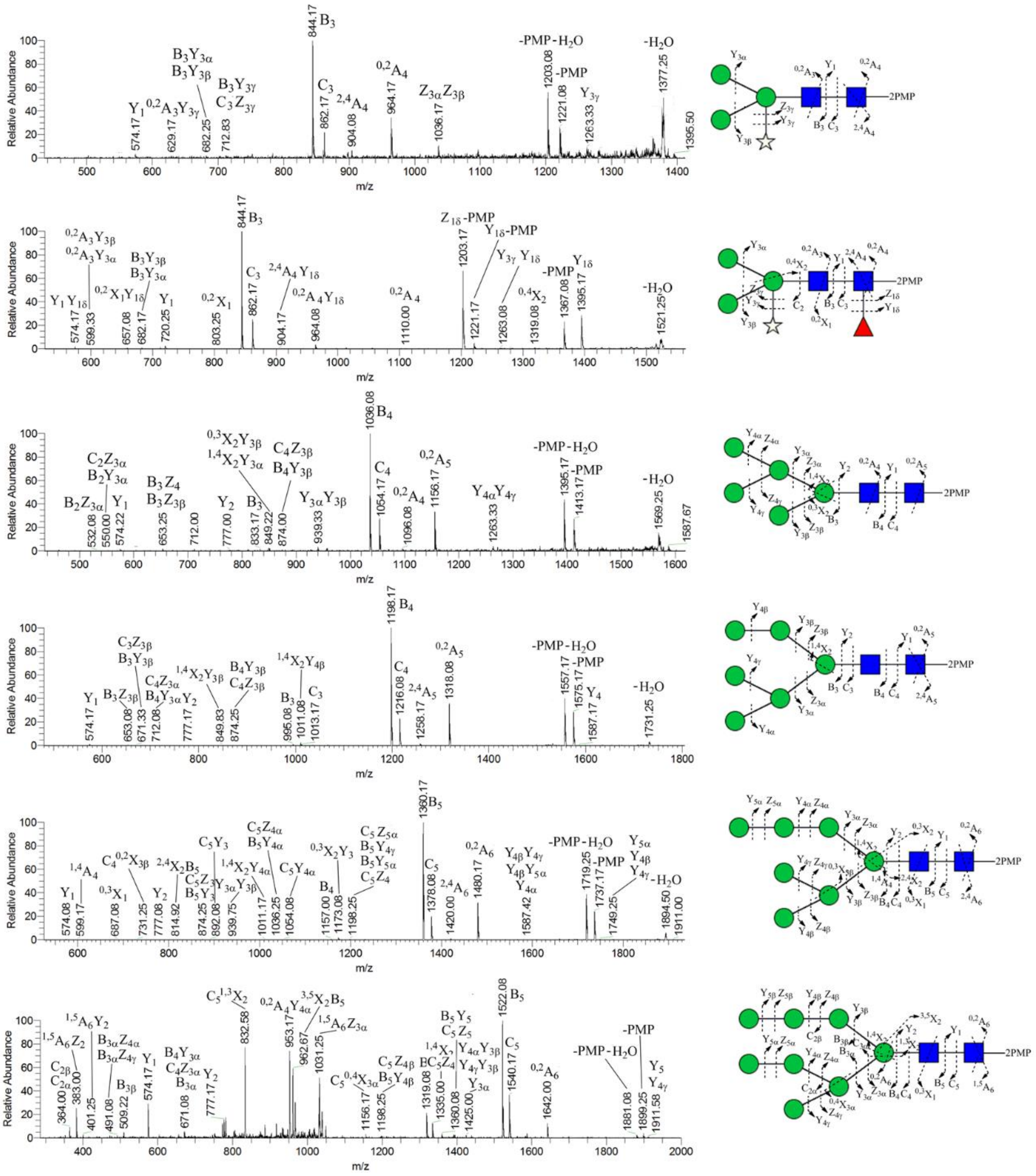

Figure S3 MS/MS analysis of *N*-glycans released from Gly Bd 30K

Symbol nomenclature: ■, *N*-acetylglucosamine; ●, mannose; ▲, fucose; ☆, xylose.

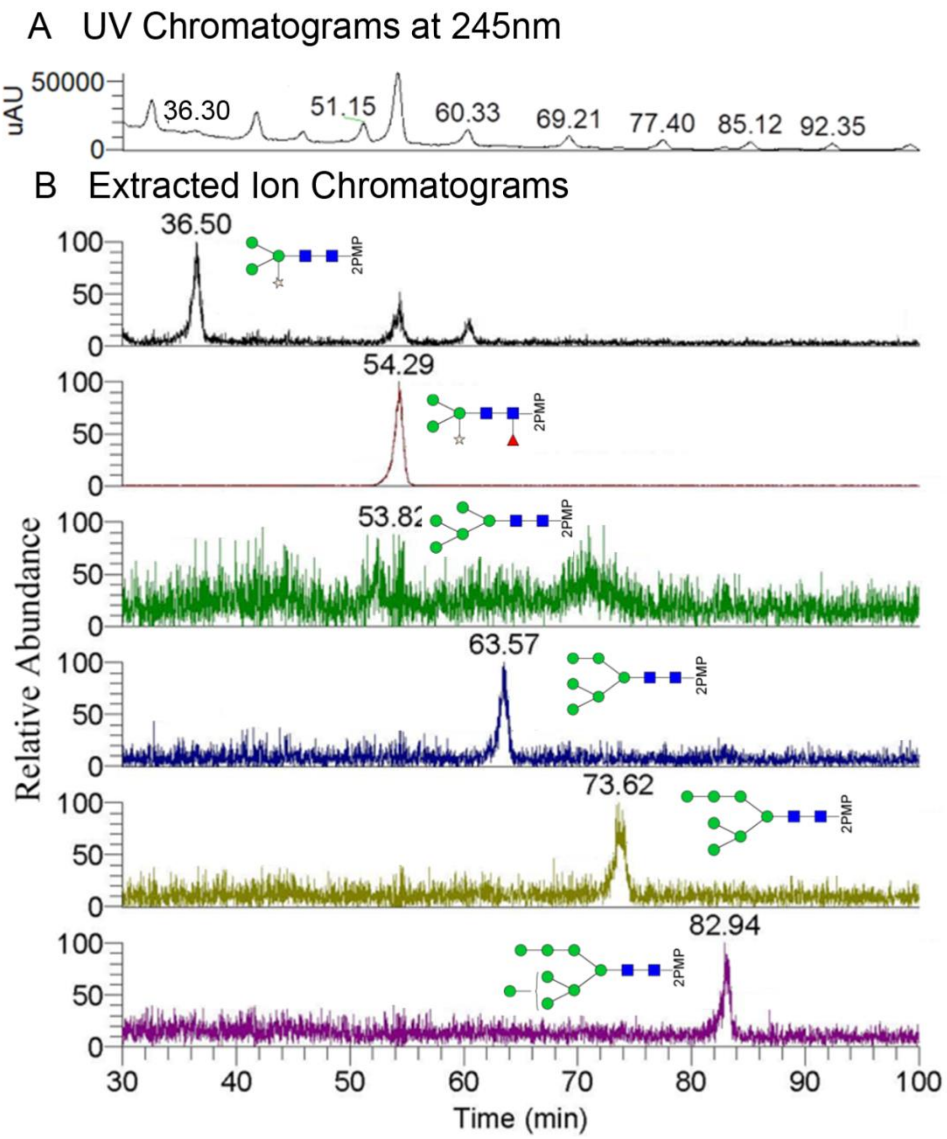

Figure S4 EICs of Gly m Bd 30K N-glycans.

A: UV chromatogram at 245 nm; chromatographic condition: solvent A, acetonitrile; solvent B, 10 mmol/L aqueous ammonium acetate (pH 6.0); time = 0 min (t = 0 min), 80% A, 20% B, 1 mL/min; t = 120 min, 60%A, 40% B, 1 mL/min. B: EICs of Gly m Bd 30K N-glycans. Symbol nomenclature: ●, mannose; ■, N-acetylglucosamine; ☆, xylos; ▲, fucose.
